# Identifying an early treatment window for predicting breast cancer response to neoadjuvant chemotherapy using immunohistopathology and hemoglobin parameters

**DOI:** 10.1186/s13058-018-0975-1

**Published:** 2018-06-14

**Authors:** Quing Zhu, Susan Tannenbaum, Scott H. Kurtzman, Patricia DeFusco, Andrew Ricci, Hamed Vavadi, Feifei Zhou, Chen Xu, Alex Merkulov, Poornima Hegde, Mark Kane, Liqun Wang, Kert Sabbath

**Affiliations:** 1 Biomedical Engineering and Radiology, Washington University in St Louis, One Brookings Drive, Mail Box 1097, Whitaker Hall 300D, St. Louis, MO 63130 USA; 2 University of Connecticut Health Center, Farmington, CT 06030 USA; 3 Waterbury Hospital, Waterbury, CT 6708 USA; 4 Hartford Hospital, Hartford, CT 06102 USA; 5 University of Connecticut, Storrs, CT 06269 USA; 6 New York City College of Technology, City University of New York (CUNY), New York, USA; 7 Department of Statistics, University of Manitoba, 186 Dysart Road, Winnipeg, Manitoba, R3T 2N2 Canada

**Keywords:** Predicting neoadjuvant chemotherapy, Personalized medicine, Near infrared imaging, Ultrasound-guided optical imaging

## Abstract

**Background:**

Breast cancer pathologic complete response (pCR) to neoadjuvant chemotherapy (NAC) varies with tumor subtype. The purpose of this study was to identify an early treatment window for predicting pCR based on tumor subtype, pretreatment total hemoglobin (tHb) level, and early changes in tHb following NAC.

**Methods:**

Twenty-two patients (mean age 56 years, range 34–74 years) were assessed using a near-infrared imager coupled with an Ultrasound system prior to treatment, 7 days after the first treatment, at the end of each of the first three cycles, and before their definitive surgery. Pathologic responses were dichotomized by the Miller-Payne system. Tumor vascularity was assessed from tHb; vascularity changes during NAC were assessed from a percentage tHb normalized to the pretreatment level (%tHb). After training the logistic prediction models using the previous study data, we assessed the early treatment window for predicting pathological response according to their tumor subtype (human epidermal growth factor receptor 2 (HER2), estrogen receptor (ER), triple-negative (TN)) based on tHb, and %tHb measured at different cycles and evaluated by the area under the receiver operating characteristic (ROC) curve (AUC).

**Results:**

In the new study cohort, maximum pretreatment tHb and %tHb changes after cycles 1, 2, and 3 were significantly higher in responder Miller-Payne 4–5 tumors (*n* = 13) than non-or partial responder Miller-Payne 1–3 tumors (*n* = 9). However, no significance was found at day 7. The AUC of the predictive power of pretreatment tHb in the cohort was 0.75, which was similar to the performance of the HER2 subtype as a single predictor (AUC of 0.78). A greater predictive power of pretreatment tHb was found within each subtype, with AUCs of 0.88, 0.69, and 0.72, in the HER2, ER, and TN subtypes, respectively. Using pretreatment tHb and cycle 1 %tHb, AUC reached 0.96, 0.91, and 0.90 in HER2, ER, and TN subtypes, respectively, and 0.95 regardless of subtype. Additional cycle 2 %tHb measurements moderately improved prediction for the HER2 subtype but did not improve prediction for the ER and TN subtypes.

**Conclusions:**

By combining tumor subtypes with tHb, we predicted the pCR of breast cancer to NAC before treatment. Prediction accuracy can be significantly improved by incorporating cycle 1 and 2 %tHb for the HER2 subtype and cycle 1 %tHb for the ER and TN subtypes.

**Trial registration:**

ClinicalTrials.gov, NCT02092636. Registered in March 2014.

## Background

The increasingly widespread use of neoadjuvant chemotherapy (NAC) in breast cancer patients has improved surgical outcomes by preoperatively downsizing the tumor volume. Since the introduction of NAC, breast-conserving surgery rates have increased [1]. Moreover, patients who have achieved pathological complete response (pCR) show improved survival rates compared with those who did not achieve pCR [2]. This relationship is so strong, in fact, that pCR is becoming a surrogate endpoint for evaluating the effectiveness of newer chemotherapy protocols [3, 4]. Early assessment of the degree of patient response to NAC can have a major impact on individualized treatment management [5].

The ability to identify patients with tumors that have a high likelihood of achieving a pCR before starting NAC could enable targeting a treatment plan to patients with only those tumor types. By eliminating ineffective treatment of patients unlikely to benefit, outcomes would be better, and toxicity would be reduced. Published literature shows that an increased probability of achieving pCR is correlated with high tumor grade, positive human epidermal growth factor receptor 2 (HER2) status, negative estrogen receptor (ER) status, and triple negative (TN) receptor status [6–9]. However, prediction models based on these tumor histopathological characteristics are imperfect; within and among these subgroups, the response to chemotherapy varies widely. Dual HER2 blockade with trastuzumab and pertuzumab in combination with cytotoxic chemotherapy now utilized in many HER2^+^ patients in the neoadjuvant setting results in a high pCR (16.8–66.2%) [10]. Despite this, there is a significant percentage of HER2^+^ patients who do not achieve a pCR or near pCR. Additionally, the pCR of ER^+^/HER2^–^ cancers is less robust (7.0–16.2%) and the pCR for TN cancers is 33.6–35% [11, 12].

Many ongoing investigations are exploring imaging techniques to monitor response and allow early modification of treatment in order to enhance outcomes [13]. The use of imaging as an early surrogate biomarker of response is appealing because it is noninvasive and might allow for a window of opportunity during which treatment regimens could be altered accordingly, depending on the expected response.

Conventional imaging methods, such as mammography and ultrasound (US), to monitor NAC have not been widely used to date due to their low sensitivity for monitoring NAC-treated tumors [14]. Positron emission tomography (PET)/computed tomography (CT) is more sensitive to tumor metabolic activity which has been shown to be an early indicator of treatment effectiveness for breast cancer in the neoadjuvant setting [15, 16]. Contrast-enhanced magnetic resonance imaging (MRI) is effective in predicting TN or HER2^+^ cancers, but is inaccurate for ER^+^/HER2^–^ breast cancers [17]. Both PET/CT and MRI require the injection of contrast agents and are costly for repeated use during treatment.

Optical tomography and spectroscopy using near infrared (NIR) diffused light have been explored as novel tools to predict and monitor the tumor vasculature response to NAC [18–31]. The NIR technique utilizes the intrinsic biomarker of hemoglobin contrast, which is directly related to tumor angiogenesis. Cost effectiveness, portability, and the absence of the need for contrast agents make NIR systems ideal for repeated use in clinical settings. We have reported the development of US-guided optical tomography using NIR diffused light coupled with a commercial ultrasound system (NIR/US) to improve light localization and quantification accuracy in the diagnosis of breast cancer [32, 33] and in predicting NAC response [20]. The logistic prediction models we developed utilize tumor pretreatment pathological parameters and hemoglobin content measured before NAC to predict pathological response [21]. The present study was designed to identify the best treatment window for predicting pathological response during NAC using breast cancer subtype, the pretreatment biomarker of total hemoglobin (tHb) level, and changes in tHb during early-treatment cycles 1, 2, and 3. Ultimately, effectively predicting the response to NAC by combining information from US-guided NIR with breast cancer subtype could help to individualize treatment.

## Methods

### Patients

The study protocol was approved by institutional review boards and was HIPAA compliant. Written informed consent was obtained from all patients. From March 2014 to June 2016, 28 patients were recruited at three hospitals. All had been referred for NAC to the one of three medical oncologists (PD, ST, and KS) and agreed to participate in our study. Five patients did not complete the study because of a change in their treatment plan or a desire to withdraw from the study. One patient had technically problematic baseline imaging. Data from these six patients were not included in the analysis. The remaining 22 patients (mean age 55 years, range 34–74 years) were repeatedly imaged by NIR/US prior to initiation of NAC, at the end of the first three treatment cycles during chemotherapy, and prior to definitive surgery. Of the 22 patients (Table 1), 11 were HER2^+^ of which eight were also ER^+^; six were ER^+^/HER2^–^, and five were TN. Of the 22 patients, 12 had T2 tumors, five had T1, and five had T3 tumors. Patients were treated with regimens based on their tumor biomarkers according to current clinical practice. For ER^+^/HER2^–^ tumors, patients were treated with dose-dense doxorubicin/cyclophosphamide every 2 weeks for four cycles followed by paclitaxel every 2 weeks for four cycles (ACT). The NIR/US cycle 1 to 3 measurements were performed at the end of the first three cycles before the paclitaxel started. For HER2^+^ tumors, all patients were treated with trastuzumab, pertuzumab, and docetaxel or paclitaxel with or without carboplatin (TPT) every 3 weeks for six cycles and one patient had two additional cycles of 5-flurouracil, epirubicin, and cyclophosphamide (FEC). The NIR/US cycle 1 to 3 measurements were performed at the end of the first three treatment cycles when TPT was given. For TN tumors, three were treated with ACT, the same as ER^+^/HER2^–^ patients, and two were treated with carboplatin and paclitaxel every 3 weeks for six cycles because of their BRCA1 gene mutation. One elderly ER^+^/HER2^–^ patient was treated with cyclophosphamide/docetaxel without doxorubicin (TC) every 3 weeks for four cycles.

**Table 1:** Patient information, tumor characteristics, Miller-Payne Grade, initial tumor size (MRI/PET and US), and treatment regimen

Age	Tumor type	NS	Mitotic count/10 HPF	TN	HER2/ER	Miller-Payne grade	Tumor size (MRI/PET)	Tumor size (US)	Treatment regimen
59	IDC	6	2	–	−/+	3	2.7	1.7	ACT
55	ILC	6	1	–	−/+	4	8.6	–^a^	ACT
33	IDC	4	2	–	−/+	3	1.6	1.5	ACT
61	IDC/ILC	6	1	+	−/−	1	3.3	1.9	ACT
59	IDC	6	5	–	−/+	2	5.6	2.2	ACT
68	IDC	6	3	–	−/+	3	7.6	2.2	ACT
51	IDC	9	40	+	−/−	5	3.6	2.2	ACT
53	IDC	9	62	+	−/−	3	PET: 3.7	4.3	ACT
									TPT
51	IDC	7	15	–	+/+	5	N/A	1.2	TPT
74	IDC	8	8	–	+/−	5	6.2	–^a^	TPT
57	IDC	9	16	–	+/−	4	6.9	–^b^	TPT&FEC
									TPT
51	IDC	9	14	–	+/−	5	3.0	1.9	TPT
59	IDC	7	1	–	+/+	5	N/A	0.6	TPT
61	IDC	4	3	–	+/+^c^	5	2.0	1.4	TPT
37	IDC/ILC	8	9	–	+/+	4	3.6	1.9	TPT
54	IDC	8	20	–	+/+	5	2.9	2.3	TPT
40	IDC	8	15	–	+/+	5	2.2	2.1	TPT
62	IDC	9	12	–	+/+	3	PET: 1.8	1.2	TPT
37	IDC	8	42	–	+/+^c^	5	1.5	1.6	TPT
72	IDC	9	42	–	−/+	3	4.3	2.3	TC
57	IDC	9	14	+	−/−	5	2.3	1.3	Carbo/T
41	IDC	9	8	+	−/−	3	4.0	4.0	Carbo/T

^a^Not US visible

^b^Much larger than the size of the US transducer

^c^ER showed a weak positive result

ACT, dose-dense doxorubicin/cyclophosphamide and paclitaxel; *Carbo/T*, carboplatin and paclitaxel, *ER* estrogen receptor, *FEC* 5flurouracil, epirubicin, and cyclophosphamide, *HER2* human epidermal growth factor receptor 2, *IDC* invasive ductal carcinoma, *ILC* invasive lobular carcinoma, *MRI* magnetic resonance imaging, *N/A* not available, *NS* Nottingham Score (out of 9), *PET* positron emission tomography, *TC* cyclophosphamide and docetaxel, *TN* triple negative, *TPT* TP and taxane-based therapy—trastuzumab, pertuzumab and docetaxel or paclitaxel with or without carboplatin, *TPT&FEC* trastuzumab, pertuzumab, paclitaxel; 5flurouracil, epirubicin, cyclophosphamide, *US* ultrasound

The HER2^+^ cohort was monitored at an additional time point of 7 days after the first treatment, and one TN and four ER^+^/HER2^–^ patients were also monitored at an optional time point of 7 days after the first treatment. The median from 7 days was 0 with a range of 0 to 2 days. Moreover, four TN and five ER^+^/HER2^–^ patients were monitored at an additional time point at the end of cycle 5. Thus, a total of 16 patients had an additional time point at 7 days after the first treatment and nine patients had an additional time point at the end of cycle 5.

All 22 patients were studied after their diagnostic core biopsy with an average interval of 28 days (median 26 days, range 7–56 days). Among the 22 patients, one had pretreatment NIR measurements 7 days after biopsy and the remaining patients had the NIR measurements more than 10 days after biopsy. All patients received the first cycle of NAC after the initial NIR/US study (median 2 days, range 0–10 days). The average interval between post-treatment NIR/US and surgery was 19 days (median 15 days, range 2–67 days). During the treatment, the NIR/US scans were performed before their scheduled chemotherapy (median 0 days, range 0–5 days). Among the 22 patients, 18 patients had pretreatment MRI and 12 patients had post-treatment MRI. Two patients had pretreatment PET.

The histologic type of 19 patients was invasive ductal carcinoma; one patient had invasive lobular carcinoma, and the other two patients had invasive mammary carcinoma with mixed ductal and lobular features. One of the 22 patients had two distinct tumor masses in the same breast, adjacent to each other and with the same histologic characteristics. For this patient, one of the two masses was used for data analysis. Invasive carcinoma within the pretreatment core biopsies was graded using the Nottingham histologic score (NS). ER, progesterone receptor (PR), and HER-2/neu (c-erbB-2) immunohistochemistry was performed on formalin-fixed, paraffin-embedded core biopsy tissue. The ER and PR were scored by a modified San Antonio scoring system [34], where the total score ranges from 0 to 8 (scores 0–2 are negative, a score of 3 is equivocal and scores ≥ 4 are positive). Testing for the HER2 gene was performed by immunohistochemistry and by gene amplification utilizing the fluorescence in situ hybridization (FISH) technique, and the results were reported in accordance with 2014 ASCO/CAP guidelines [35]. Results were reported as equivocal HER2 if there was weak to moderate, incomplete membranous staining in > 10% of cells or if FISH showed a HER2/CEP17 ratio < 2, or if the HER2 copy number was ≥ 4 and < 6. HER2 results were negative if the immunohistochemistry or FISH assays fell below the thresholds for interpretation as equivocal. All assays were performed on pretreatment core biopsy samples.

### Pathology assessment

Pathologic response was assessed by applying the Miller-Payne grading criteria to definitive surgery specimens in comparison with initial core biopsies (Table 1). Two breast pathologists (AR and PH) individually evaluated cases from their respective hospitals and additional cases from the third hospital. The Miller-Payne system [36] divides pathologic response into five grades based on a comparison of tumor cellularity between the pretreatment core biopsy and the definitive surgical specimen. Grade 1 indicates no change or some minor alteration in individual malignant cells but no reduction in overall cellularity; this is a pathological nonresponse (pNR). Grade 2 indicates a minor loss of tumor cells (up to 30%) but with overall cellularity still high; this is a partial pathologic response (pPR). Grade 3 indicates an estimated 30–90% reduction in tumor cells (pPR). Grade 4 indicates a marked disappearance of tumor cells (> 90%), with only small clusters or widely dispersed individual cells remaining (almost pCR). Grade 5 indicates that no malignant cells are identifiable in sections from the tumor bed (pCR). Grade 5 may show that necrosis, granulation tissue, histiocytes, and vascular fibroelastotic stroma remains, often containing macrophages. Residual ductal carcinoma in situ (DCIS) is acceptable for Miller-Payne grade 5.

### US and NIR system and imaging

Ultrasound examinations were performed using a commercial ultrasound system (Phillips IU22 or GE Logiq 5) at the corresponding hospital. Three NIR systems with identical designs were used at the three hospitals, and the details have been given previously [24]. Briefly, the NIR/US probe consists of the commercial US transducer located centrally, with source and detector light guides (optical fibers) distributed around the periphery of the NIR/US probe. Four laser diodes of 740 nm, 780 nm, 808 nm, and 830 nm optical wavelengths were sequentially switched to nine positions on the probe, while the reflected light was coupled by the light guides to 14 parallel detectors. The entire NIR data acquisition interval was less than 5 s. For each patient, US images and optical measurements were acquired simultaneously in the cancer region and a normal region within the contralateral breast in the same quadrant as the cancer. At each cancer and normal region, multiple datasets were acquired. The optical data acquired from the normal contralateral breast was used as a reference for calculating the background optical absorption and reduced scattering coefficients that were used in the image reconstruction of the lesions.

Details of the optical imaging reconstruction algorithm with experimental validation have been described elsewhere [37]. Briefly, the NIR reconstruction takes advantages of the ultrasound localization of lesions to segment the imaging volume into a region of interest (ROI) and background nonlesion regions. Since the spatial resolution of diffused light is poorer than that of US, the ROI is chosen to be at least two to three times larger than the tumor size measured by coregistered US in *x* to *y* dimensions. In addition, because the depth localization of diffused light is very poor, a tighter ROI in the depth dimension is set by using coregistered US. For each patient, the same size of ROI as that obtained from the pretreatment US is used for processing all datasets obtained at different treatment cycles. Therefore, the changes in tumor size seen by US during treatment have no major effect on the NIR image reconstruction. Among the 22 patients, two patients had initially palpable tumors with ill-defined and heterogeneous pretreatment ultrasound images. For these patients, tumor sizes estimated from pretreatment MRI measurements were used to assist in determining the US ROI in the *x* to *y* dimensions. The ROI in the depth dimension was typically set from the top border of the ill-defined tissue pattern to the chest wall as seen by US.

The optical absorption distribution at each wavelength was reconstructed and the tHb concentration, oxygenated hemoglobin (oxyHb) concentration, and deoxygenated hemoglobin (deoxyHb) concentration maps were computed from absorption maps at the four wavelengths [38]. The maximum values of tHb, oxyHb, and deoxyHb were measured for each set of hemoglobin maps. For each patient imaged at each time point, an average maximum that was obtained from 5 to 10 quality NIR images at the tumor location was used to characterize the tumor. Data with patient motion as evaluated by using two coregistered US images before and after each NIR measurement were excluded from averaging. To assess the response of each patient, the tHb obtained before treatment was taken as the baseline and the percentage normalized to the baseline (%tHb) was used to quantitatively evaluate the remaining tumor vascular fraction during chemotherapy.

### MRI and US imaging and measurements

Nineteen patients had well-defined tumors visible by US. Tumor sizes were measured by US technologists under direct supervision of attending radiologists. The percentage ratio (%US) of the largest dimension of each post-treatment measurement over the largest dimension of pretreatment measurement was used to evaluate the morphological change during NAC. One patient had a much larger tumor size than the US transducer, and the baseline US measurement was not accurate. The initial tumor mass of this patient measured by MRI was 6.9 cm. US scans performed from both medial lateral and cranial-caudal directions using the 5-cm US transducer were used to estimate the approximate mass center. Then the combined probe of 10-cm diameter was placed at the estimated mass center for NIR data acquisition. This procedure had minimal effect on NIR reconstruction. For the two patients with initially palpable but ill-defined and heterogeneous US images, the tumor location at each measurement was tracked using previous US images as references. The tumor clock position, distance of the tumor from the nipple, and depth below the skin were documented for each case. Additionally, the tumor posterior shadowing and surrounding tissue structures, as well as the metal clip position, were also reviewed and used to help identify the tumor for each subsequent measurement. If an MRI was ordered for clinical reasons, the MRI measurements were obtained from the medical records of the patients.

### Prediction models

We have previously developed a logistic regression model to predict the NAC response of a patient using pretreatment tumor clinicopathologic variables, tumor subtype, and baseline tHb values [21]. Briefly, logistic regression is a statistical modeling approach that can be used to describe the relationship of several predictor variables X_1,_ X_2_… X_k_ to a dichotomous response variable Y, where Y is coded 1 (responder) or 0 (nonresponder) for its two possible categories [39]. The model can be written in the form of the conditional probability of the occurrence of one of the two possible outcomes of Y, as follows:

1 $$ pr\left(Y=1\ |\ \mathrm{X}1,\mathrm{X}2,\dots \mathrm{Xk}\right)=\frac{1}{1+\exp \left(-\left(\beta 0+\sum \limits_{n=1}^k\beta nXn\right)\ \right)} $$

The estimated outputs (probability) for each set of predictor variables range from 0 to 1. Given the data on Y, X_1_, … X_k_, the unknown parameters βn, n = 0, 1, …, k_,_ *n* = 0, 1, …k can be estimated using the maximum likelihood method.

In this study, we used the data from 32 patients obtained from an earlier study as a training set [20] to estimate a total of four groups of logistic models, and validated these models using the new dataset reported in this study as a testing dataset. The earlier data obtained from 2008 to 2011 were acquired from almost identical NIR systems with the same data processing and image reconstruction procedures as reported in this study.

To validate that the early data and new data are generated from the same population, we have introduced a dummy variable X_k + 1_ in Eq. 1 which is coded as 0 for early data and 1 as new data [40, 41]. We have estimated the model with this dummy variable along with the eight predictor variables using the combined datasets. The estimate on *β*_*k* + 1_ (*P* = 0.214) is statistically insignificant. Therefore, we can assume both datasets come from the same patient population.

The Matlab (version 2016a) logistic regression function glmfit was used to estimate the coefficients *β*_*n*_*βn*, *n* = 0, 1, …, *k*, and glmval was used to calculate the receiver operating characteristic (ROC) with these coefficients for the training set. The same coefficients obtained from the training set were used to predict the response for the testing set.

### Evaluation of prediction models

We also assessed the overall performance of the prediction models through the ROC curves and the area under the curves (AUCs) for each pair of training and testing sets of each prediction model. The early data used for training had 20 ER^+^/HER2^–^ patients, six TN patients, five HER2^+^ patients, and one ER^–^/PR^+^/HER2^–^ patient [20]. Similar to the new patient cohort, ER^+^/HER^–^ (*n* = 14) and TN (*n* = 6) tumors were treated with ACT every 2 weeks for eight cycles. Six ER^+^/HER2^–^ tumors and one ER^–^/PR^+^/HER2^–^ tumor were treated with ACT (*n* = 3) or TC (*n* = 4) every 3 weeks for six cycles. HER2^+^ tumors (*n* = 5) were treated with trastuzumab and docetaxel or paclitaxel with or without carboplatin (TPT) every 3 weeks for six cycles.

Since the early data did not contain any patients treated with dual HER2 blockade, we have randomly selected six HER2^+^ patients treated with this regimen from the current study and added these six datasets to the training data. For each random selection, the total of patients in training was 38 (32 from the early data and 6 from this study) and testing was 16 from this study. A total of 6 out of 11 random selections result in 462 combinations of paired training and testing datasets for each group of predictors, and the mean of 462 AUC values was used to evaluate the training and testing results of each prediction model. Each pair of training and testing ROCs was generated using a threshold of 0.5. This was used to separate responders (> 0.5) and nonresponders (≤ 0.5) for each prediction model output. We also used the 462 AUC values to construct the 95% confidence interval (CI) for the mean AUC for each model using a binomial formula. These confidence intervals can provide summary information on comparisons of the different models in terms of their AUC values. For example, if model I has a higher mean AUC than model II, and if their corresponding confidence intervals do not overlap, then this is an indication that model I may have a higher prediction power than the model II in terms of the AUC criterion. However, this interpretation should be understood with the caveat that the 462 values are not true random samples.

### Selection of predictors

To select the independent predictors, Spearman’s rho was evaluated between each predictor and Miller-Payne grade and between each pair of predictors. Spearman’s rho is more appropriate for assessing the relationship for both continuous and discrete variables. Note that both training and testing data were combined to assess the predictors and the Spearman’s rhos reported in this section are from the entire cohort of both earlier and new data (Table 2). To compute rho, the tumor HER2, ER, and TN status were coded as: 1 for TN and 0 for otherwise; 1 for HER2^+^ and 0 for HER2^–^; and 0 for ER^+^ and 1 for ER^–^. Note that 1 presents increased probability of achieving pCR and 0 otherwise.

**Table 2:** Spearman’s rho correlation coefficient and *P* value between Miller-Payne grade and tumor pathological variables (MC, NS), tumor subtype (HER2, ER, TN), tHb, and %tHb measured at the end of cycles 1 to 3

	NS	MC	HER2	ER	TN	tHb	%tHb cycle 1	%tHb cycle 2	%tHb cycle 3
rho	0.42	0.45	0.45	0.29	0.12	0.48	0.50	0.52	0.69
*P*	*P* = 0.001	*P* < 0.001	*P* < 0.001	*P* = 0.035	*P* = 0.364	*P* < 0.001	*P* < 0.001	*P* < 0.001	*P* < 0.001

Data are from [20] and this study (*n* = 54 patients)

*ER* estrogen receptor, *HER2* human epidermal growth factor 2, *MC* mitotic count, *NS* Nottingham score, *tHb* total hemoglobin, *TN* triple negative

Both HER2 and ER are highly correlated with Miller-Payne grade (rho = 0.45, *P* < 0.001; rho = 0.29, *P* = 0.035) and are independent of each other (rho = 0.01, *P* = 0.928); thus, they were selected as predictors. TN tumors did not have a significant correlation with Miller-Payne grade (rho = 0.124, *P* = 0.362). However, TN was selected as a predictor because it is used clinically to characterize this group of patients. Among tumor pathological parameters, NS is a traditional pathological variable used by oncologists to predict response. NS is highly correlated with mitotic counts (rho = 0.82, *P* < 0.001). Thus, only NS was selected as an independent pathological predictor and used in each HER2, ER, and TN subtypes to predict response.Baseline tHb and %tHb changes measured during first three treatment cycles are highly correlated with Miller-Payne grade ( see Table 2) and were selected to assess the optimal time window to predict response.

### Statistical analysis

A two-sample two-sided *t* test was used to calculate the statistical significance for comparisons between responder groups, and a difference with a *P* value of 0.05 was considered significant. The *t* test was also used to test the difference between AUCs of different prediction models when their 95% CIs overlapped. Minitab 17 software (Minitab, State College, PA) was used for statistical calculations.

## Results

**Fig. 1:**
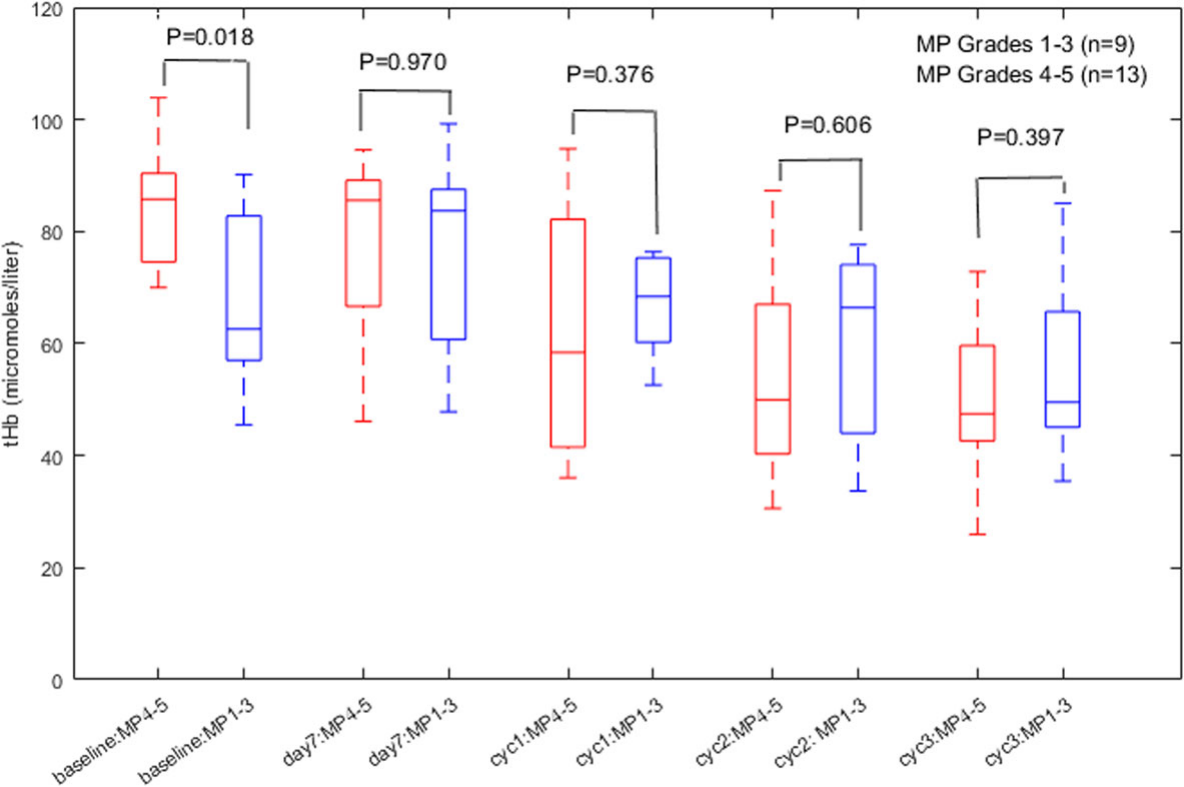
Box plot of mean maximum total hemoglobin (tHb; μmol/L) of two responder groups of Miller-Payne (MP) 4–5 and MP 1–3 measured at baseline, day 7, and at the end of cycles (cyc) 1, 2, and 3 of NAC

There were nine Miller-Payne grade 1–3 tumors and 13 grade 4–5 tumors. For grade 4–5 tumors, the pretreatment mean maximum tHb was 84.8 ± 11.3 μmol/L (mean ± standard deviation), whereas for grade 1–3 tumors the pretreatment mean maximum tHb was 67.9 ± 16.2 μmol/L (*P* = 0.018). The mean difference of the maximum tHb was 16.9 μmol/L and the 95% CI was 3.5–3.04 μmol/L. However, no statistical significance was found at day 7 and at the end of treatment cycles 1, 2, and 3 as the mean tHb level was reduced in grade 4–5 tumors but the mean level did not change in grade 1–3 tumors (Fig. 1).

**Fig. 2:**
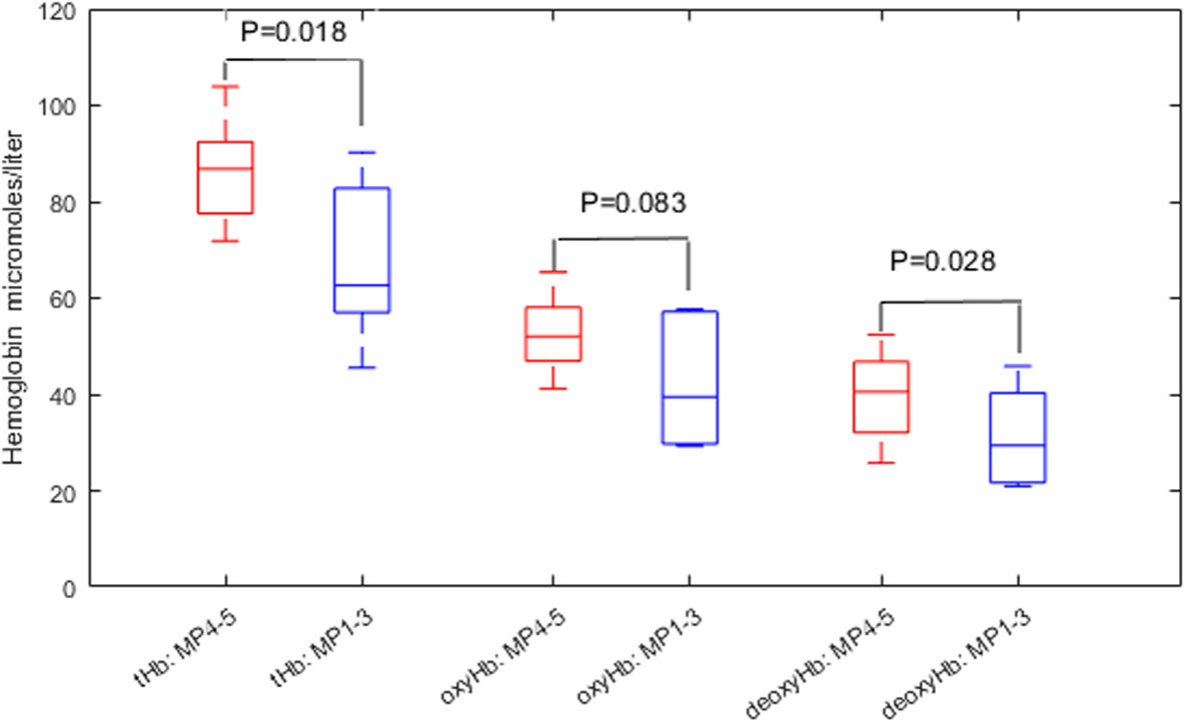
Box plot of pretreatment maximum total hemoglobin (tHb), oxygenated hemoglobin (oxyHb), and deoxygenated hemoglobin (deoxyHb) (μmol/L) of two responder groups. MP, Miller-Payne

The pretreatment oxyHb was not significantly different between grade 4–5 and grade 1–3 tumors (*P* = 0.083); however, the deoxyHb difference was significant (*P* = 0.028) (Fig. 2). In subsequent measurements, no significant difference was observed in either oxyHb or deoxyHb.

**Fig. 3:**
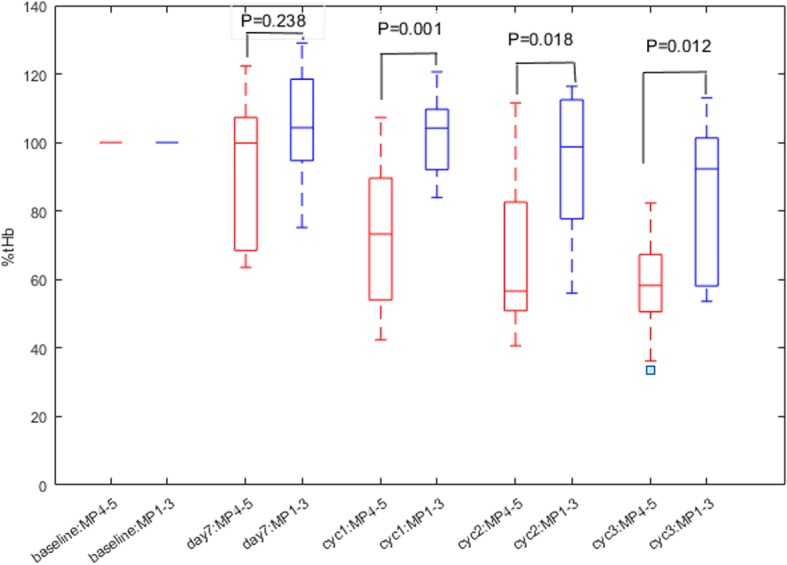
Box plot of percent total hemoglobin (%tHb) of two responder groups of Miller-Payne (MP) 4–5 and MP 1–3 measured at baseline, day 7, and at the end of cycles (cyc) 1, 2, and 3 of NAC

The %tHb (percentage fraction from baseline) was calculated and the results for the two groups are given in Fig. 3. No statistical significance between the two groups was found at day 7 (*P* = 0.238). Statistical significance was achieved at the end of cycle 1. For Miller-Payne grade 1–3 tumors %tHb was 102 ± 12%, whereas for grade 4–5 tumors %tHb was 72 ± 22%. The mean difference was 30% (*P* = 0.001), and the 95% CI was 14.6–45.0%. The significance remained high at the end of cycles 2 and 3, with mean differences of 26% (*P* = 0.018) and 95% CI 5.1–46.4%, and 25% (*P* = 0.012) and 95% CI 6.4–43.7%, respectively.

In this new cohort, Spearman’s rhos calculated between Miller-Payne grade and pretreatment tHb, oxyHb, and deoxyHb, as well as %tHb measured at different cycles, reveal that the pretreatment maximum tHb and maximum deoxyHb have achieved statistical significance (*P* = 0.049 and 0.030; Table 3). The %tHb values measured at the end of cycles 1, 2, and 3 are highly predictive (*P* = 0.002, *P* = 0.006, and *P* = 0.048, respectively), while %tHb measured at day 7, the end of cycle 5, and before operation are not predictive (*P* = 0.321, *P* = 0.321, and *P* = 0.202, respectively).

**Table 3:** Spearman’s rho correlation coefficient and *P* value between Miller-Payne grade and pretreatment tHb (maximum), oxyHb (maximum), deoxyHb (maximum), %tHb at day 7, and at the end of cycles 1, 2, 3, and 5, and before surgery

	tHb (max)	oxyHb (max)	deoxyHb (max)	%tHb day7	%tHb cycle 1	%tHb cycle 2	%tHb cycle 3	%tHb Cycle 5	%tHb before surgery
rho	0.43	0.28	0.46	0.26	0.61	0.57	0.43	0.26	0.29
*P*	*P* = 0.049	*P* = 0.205	*P* = 0.030	*P* = 0.321	*P* = 0.002	*P* = 0.006	*P* = 0.048	*P* = 0.321	*P* = 0.202

Data are from the new cohort (*n* = 22 patients)

*deoxyHb* deoxygenated hemoglobin, *oxyHb* oxygenated hemoglobin, *tHb* total hemoglobin

There is no correlation between the pretreatment tumor size measured by MRI (*n* = 18, *P* = 0.150) and US (*n* = 19, *P* = 0.152) and the Miller-Payne grade.

**Fig. 4:**
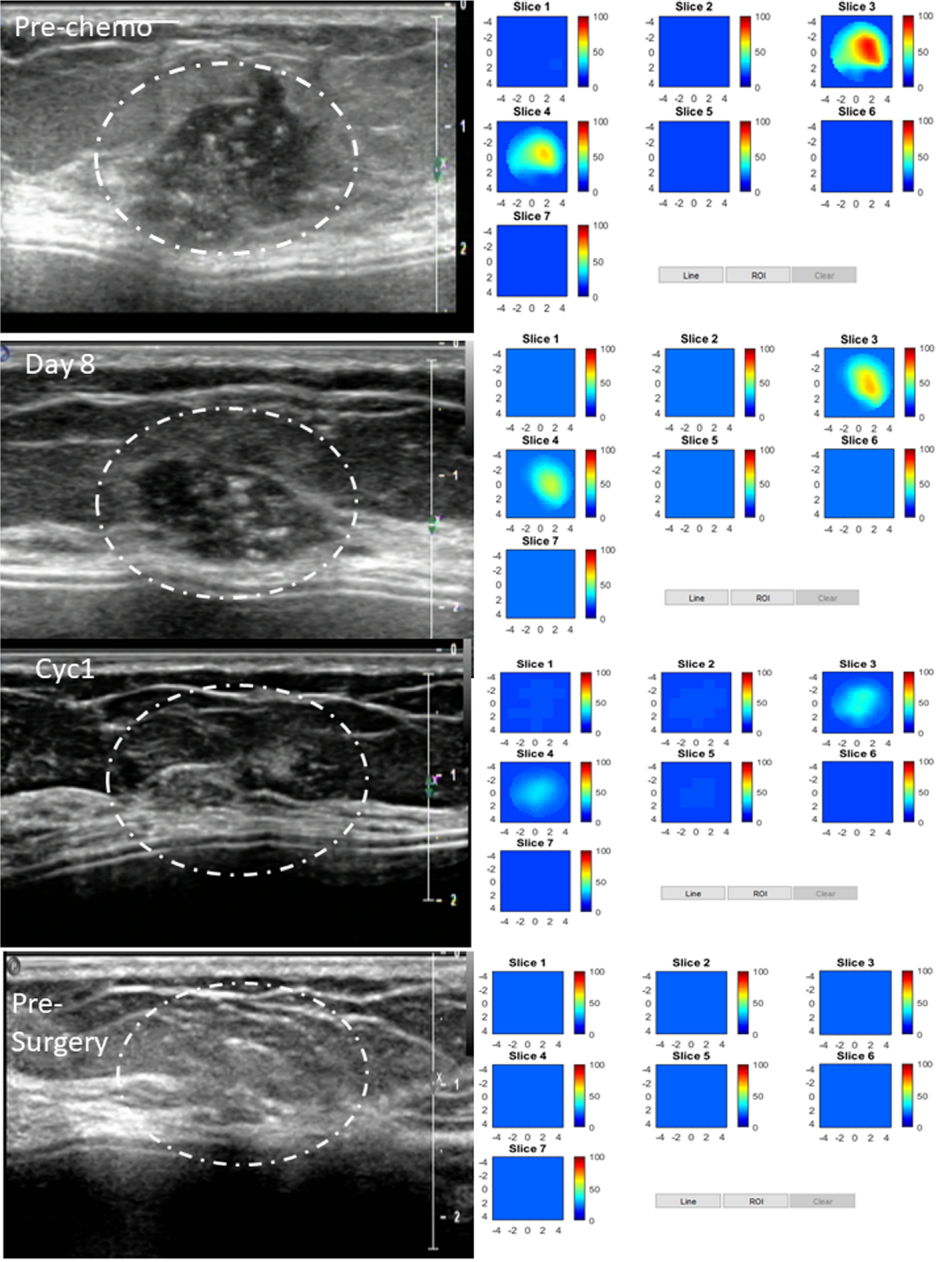
pCR in an HER2-positive tumor in a 51-year-old woman with a high-grade invasive ductal carcinoma treated with TPT every 3 weeks for six cycles. Left panel: US images obtained at pretreatment, at day 7, at the completion of cycle 1, and before surgery. Right panel: tHb concentration maps obtained at the corresponding time points. Each map shows seven subimages marked as slice 1 to 7, and each subimage shows spatial *x* and *y* distribution of tHb concentration reconstructed from 0.5 cm to 3.5 cm below the skin surface. The depth spacing between the subimages in depth is 0.5 cm. The color bar is tHb in micromoles per liter. This patient had a complete pathologic response with no residual tumor, Miller-Payne grade 5

An example of a pCR is shown in an HER2-positive tumor (Fig. 4) in a 51-year-old woman with a high-grade invasive ductal carcinoma treated with TPT every 3 weeks for six cycles. US images obtained at pretreatment, day 7, at the completion of cycle 1, and before surgery are shown in the left panel. The tumor was well defined and seen by US before treatment and at day 7, was barely visible at the completion of cycle 1, and was not visible at the completion of cycles 2 (data not shown) and 3 (data not shown), and before surgery. tHb concentration maps obtained at the corresponding time points are shown in the right panel. The tHb reduced from 85.8 μmol/L measured before treatment to 69.4, 36.3, and 21.8 μmol/L measured at day 7, before the completion of cycle 1, and before surgery, respectively. tHb measured at the completion of cycles 2 and 3 were 41.8 and 28.7 μmol/L, respectively (data not shown). A dramatic tHb reduction occurred at the end of cycle 1 (%tHb = 42%). This patient had a complete pathologic response with no residual tumor (Miller-Payne grade 5).

**Fig. 5:**
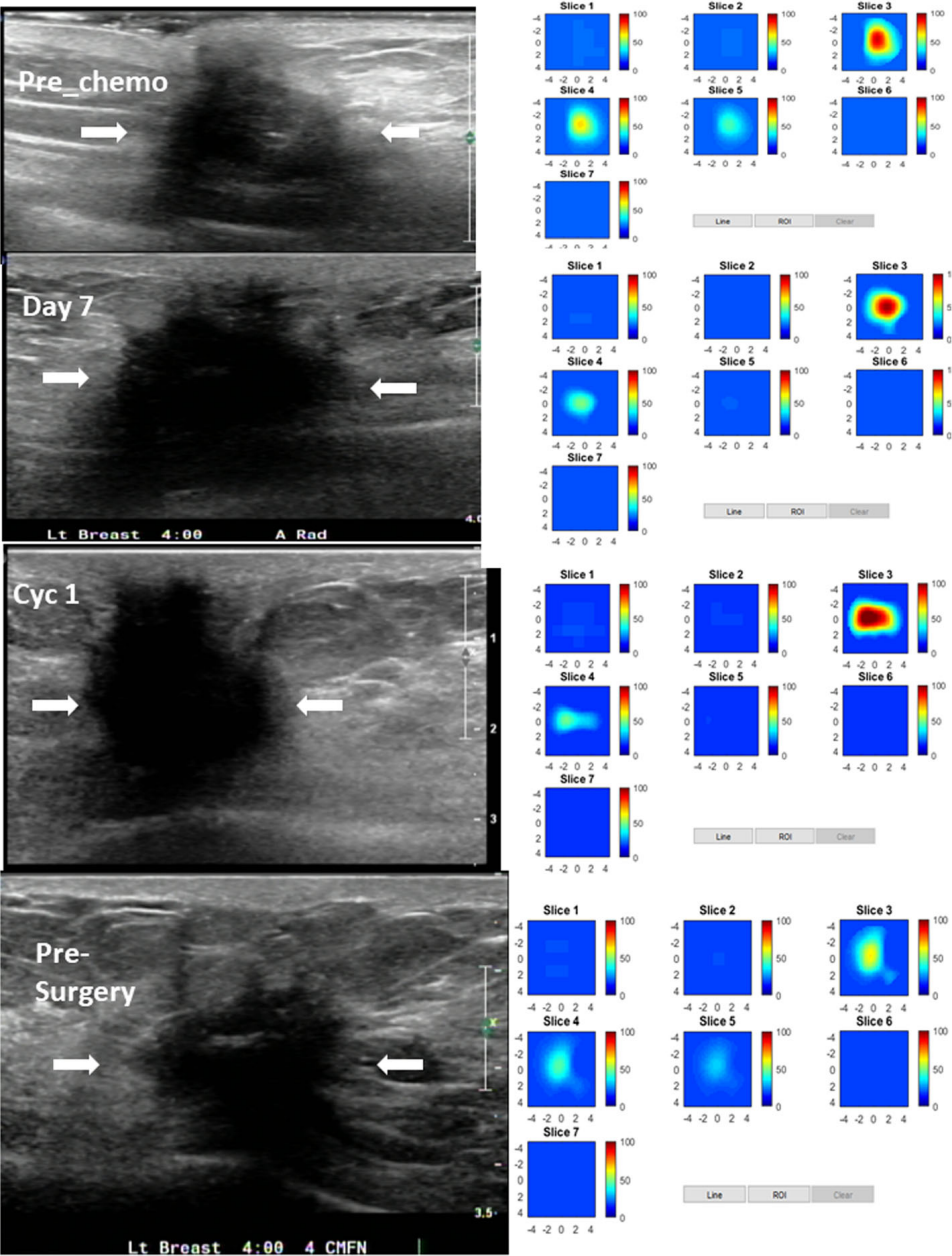
Partial response in a high-grade ER-positive/HER2-negative tumor in a 72-year-old woman with a locally recurrent invasive ductal carcinoma. She was treated with cyclophosphamide and docetaxel every 3 weeks for four cycles before surgery. Left panel: US images at four time points of pretreatment, at day 7, at the completion of cycle (Cyc) 1, and before surgery. The tumor was ill-defined with an unclear boundary seen by US. Right panel: tHb concentration maps obtained at the corresponding time points. The patient had a partial response with residual invasive carcinoma of 2.8 cm, Miller-Payne grade 3

An example of a partial responder is shown in a high-grade ER-positive/HER2-negative tumor (Fig. 5). A 72-year-old woman had a locally recurrent invasive ductal carcinoma. She was treated with TC every 3 weeks for four cycles before surgery. US images at the four time points pretreatment, day 7, at the completion of cycle 1, and before surgery are shown in the left panel. The tumor was ill-defined with unclear boundary seen by US. tHb concentration maps obtained at the corresponding time points are shown in the right panel. tHbs of 90.2, 99.3, 108.8, 105.0 (data not shown), 85.0 (data not shown), and 69.0 μmol/L were measured at pretreatment, day 7, at the completions of cycles 1 to 3, and before surgery, respectively. The patient had a partial response with a residual invasive carcinoma of 2.8 cm (Miller-Payne grade 3).

**Fig. 6:**
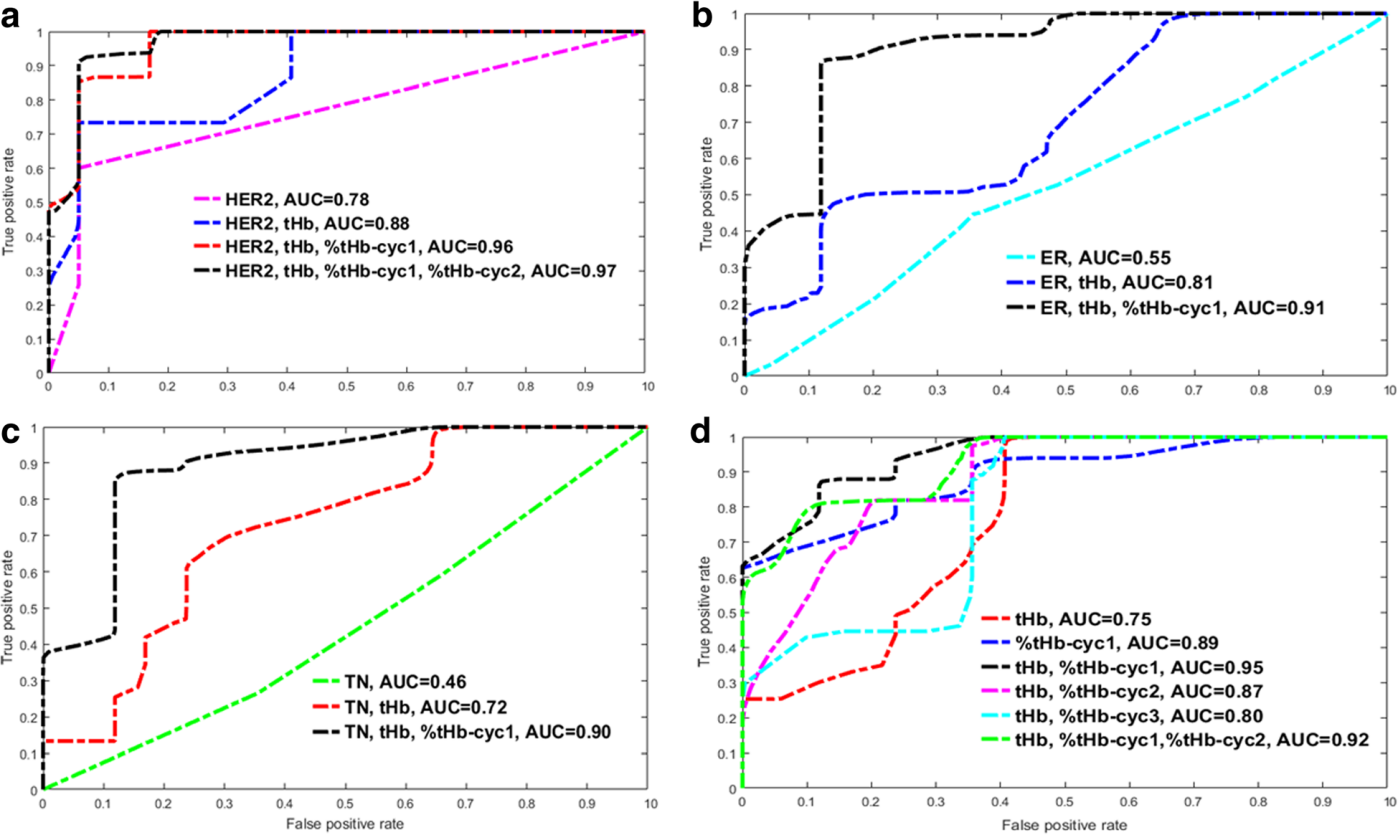
ROC curves of testing data of four groups of prediction models based on **a** HER2 subtype and hemoglobin parameters, **b** ER subtype and hemoglobin parameters, **c** TN subtype and hemoglobin parameters, and **d** hemoglobin parameters

Based on tumor biomarkers and hemoglobin measurements, predictors are grouped into four categories:1) HER2 status with hemoglobin predictors; 2) ER with hemoglobin predictors; 3) TN with hemoglobin predictors; and 4) hemoglobin predictors (see Table 4). ROCs of validation or testing of these four groups are given in Fig. 6 and Table 4. Note that predictor groups that achieve higher training AUCs do not necessarily translate into higher testing AUCs, and higher AUCs of testing data are used to compare prediction models.

**Table 4:** Four groups of logistic regression models based on tumor subtype and hemoglobin parameters, AUC of training data, and AUC of testing data

Tumor subtypes	Training AUC (95% CI)	Testing AUC (95% CI)
Group 1 (HER2 subtype)		
Her2	0.71 (0.66–0.75)	0.78 (0.74–0.81)
Her2, tHb	0.88 (0.85–0.91)	0.88 (0.85–0.91)
Her2, tHb, ER	0.91 (0.89–0/94)	0.87 (0.84–0.90)
Her2, tHb, NS	0.91 (0.88–0.94)	0.85 (0.82–0.88)
Her2, tHb, %tHb_cyc1	0.89 (0.87–0.92)	**0.96 (0.95**–**0.98)**
Her2, tHb, %tHb_cyc2	0.89 (0.86–0.91)	0.94 (0.92–0.96)
Her2, tHb, %tHb_cyc3	0.96 (0.94–0/97)	0.89 (0.86–0.92)
Her2, tHb, %tHb_cyc1, %tHb_cyc2	0.90 (0.87–0.93)	**0.97 (0.96**–**0.99)**
Her2, tHb, %tHb_cyc1, %tHb_cyc3	0.96 (0.94–0.97)	0.88 (0.85–0.91)
Her2, tHb, %tHb_cyc2, %tHb_cyc3	0.96 (0.94–0.98)	0.88 (0.85–0.91)
Her2, tHb, %tHb_cyc1, %tHb_cyc2, %tHb_cyc3	0.96 (0.94–0.98)	0.88 (0.86–0.92)
Group 2 (ER subtype)		
ER	0.67 (0.63–0.72)	0.55 (0.50–0.59)
ER, tHb	0.81 (0.77–0.85)	0.69 (0.64–0.73)
ER, tHb, NS	0.83 (0.80–0.87)	0.69 (0.65–0.73)
ER, tHb, %tHb_cyc1	0.85 (0.82–0.89)	**0.91 (0.88**–**0.93)**
ER, tHb, %tHb_cyc2	0.86 (0.83–0.89)	0.79 (0.75–0.83)
ER, tHb, %tHb_cyc3	0.97 (0.95–0.98)	0.77 (0.73–0.81)
ER, tHb, %tHb_cyc1, %tHb_cyc2	0.88 (0.85–0.91)	0.86 (0.83–0.89)
ER, tHb, %tHb_cyc1, %tHb_cyc3	0.96 (0.95–0.98)	0.76 (0.73–0.80)
ER, tHb, %tHb_cyc2, %tHb_cyc3	0.97 (0.95–0.98)	0.77 (0.73–0.81)
Group 3 (TN subtype)		
TN	0.55 (0.51–0.56)	0.46 (0.41–0.50)
TN, tHb	0.77 (0.74–0.81)	0.72 (0.68–0.76)
TN, tHb, NS	0.81 (0.78–0.85)	0.69 (0.65–0.75)
TN, tHb, %tHb_cyc1	0.84 (0.81–0.87)	**0.90 (0.87**–**0.93)**
TN, tHb, %tHb_cyc2	0.85 (0.82–0.88)	0.84 (0.81–0.88)
TN, tHb, %tHb_cyc3	0.96 (0.94–0.98)	0.76 (0.72–0.80)
TN, tHb, %tHb_cyc1, %tHb_cyc2	0.85 (0.82–0.88)	0.90 (0.87–0.93)
TN, tHb, %tHb_cyc1, %tHb_cyc3	0.96 (0.94–0.98)	0.75 (0.71–0.79)
TN, tHb, %tHb_cyc2, %tHb_cyc3	0.96 (0.94–0.98)	0.75 (0.71–0.79)
Group 4 (tHb and %tHb, all patients)		
tHb	0.77 (0.73–0.81)	0.75 (0.71–0.79)
tHb, %tHb_cyc1	0.83 (0.80–0.87)	**0.95 (0.93**–**0.97)**
tHb, %tHb_cyc2	0.84 (0.80–0.86)	0.87 (0.84–0.90)
tHb, %tHb_cyc3	0.94 (0.92–0.97)	0.80 (0.76–0.84)
tHb, %tHb_cyc1, %tHb_cyc2	0.84 (0.81–0.88)	0.92 (0.90–0.95)
tHb, %tHb_cyc1, %tHb_cyc2, %tHb_cyc3	0.94 (0.92–0.96)	0.80 (0.76–0.84)
%tHb_cyc1	0.79 (0.75–0.83)	0.89 (0.86–0.92)
%tHb_cyc2	0.83 (0.80–0.86)	0.81 (0.78–0.85)
%tHb_cyc3	0.94 (0.92–0.96)	0.82 (0.78–0.85)
%tHb_cyc1, %tHb_cyc2	0.82 (0.78–0.87)	0.87 (0.83–0.90)
%tHb_cyc1, %tHb_cyc2, %tHb_cyc3	0.94 (0.92–0.96)	0.82 (0.79–0.86)

Bold entries indicate the best set of predictors in each group

*AUC* area under the curve, *CI* confidence interval, *ER* estrogen receptor, *HER2* human epidermal growth factor receptor 2, *NS* Nottingham score, *tHb* total hemoglobin, *TN* triple negative

For HER2 group 1, HER2 used alone can achieve an AUC of 0.78 (95% CI 0.74–0.81). When HER2 and tHb are used together, the AUC reached 0.88 (95% CI 0.85–0.91). The addition of ER or NS essentially produces similar AUCs of 0.87 (95% CI 0.84–0.90) and 0.85 (0.82–0.88). However, the statistical significance of HER2 and tHb is higher than HER2, tHb, and ER (*P* = 0.016) and HER2, tHb, and NS (*P* < 0.001). This suggests that HER2 and tHb are the best pretreatment predictors regardless of ER or NS. The highest AUC of 0.96 (95% CI 0.95–0.98) is achieved when %tHb measured at the end of cycle 1 is added to HER2 and tHb. This was further modestly improved to 0.97 (95% CI 0.96–0.99) when %tHb measured at end of cycles 1 and 2 are used together with HER2 and tHb. Thus, the optimal time window with an accurate prediction of pathologic response in the HER2 subtype is at the end of cycle 2.

For ER group 2, ER used alone can achieve an AUC of 0.55 (95% CI 0.50–0.59). ER and tHb achieve AUC of 0.69 (95% CI 0.64–0.73), and ER, tHb, and NS achieve AUC of 0.69 (95% CI 0.65–0.73). NS does not add any value to predicting the response of the ER patient group. The addition of %tHb measured at the end of cycle 1 improves the AUC of ER and tHb to 0.91 (95% CI 0.88–0.93). For TN group 3, TN used alone can achieve an AUC of 0.46 (95% CI 0.40–0.50). TN and tHb achieve an AUC of 0.72 (95% CI 0.68–0.76), and TN, tHb, and NS achieve an AUC of 0.69 (95% CI 0.65–0.75). NS does not add any value to predicting the response of the TN patient group. The addition of %tHb measured at cycle 1 improves the AUC to 0.90 (95% CI 0.87–0.93). For both ER and TN subtypes, %tHbs measured at the end of cycles 2 and 3 do not significantly improve prediction (Table 4). Thus, the optimal time window for assessing response in ER or TN subtypes is at the end of cycle 1.

For group 4, tHb used alone can achieve an AUC of 0.75 (95% CI 0.71–0.79), and %tHb cycle 1 used alone can achieve an AUC of 0.89 (95% CI 0.86–0.92). tHb and %tHb cycle 1 achieve an AUC of 0.95 (95% CI 0.93–0.97), which is significantly higher compared with the ER or TN subtype groups (*P* < 0.001). %tHbs measured at the end of cycles 2 and 3 do not significantly improve prediction (Table 4). Therefore, for ER and TN subtypes, tHb and %tHb are the best predictors and the early window for prediction is at the end of cycle 1.

The sensitivity, specificity, positive predictive value (PPV), and negative predictive values (NPV) of the best groups of predictors are shown in Table 5.

**Table 5:** Sensitivity, specificity, PPV, NPV, and AUC of the best set of predictors based on tumor subtype and hemoglobin parameters of the testing data

	Sensitivity	Specificity	PPV	NPV	AUC
Her2, tHb, %tHb_cyc1	73.7	94.9	92.4	84.8	0.96
Her, tHb, %tHb_cyc1, %tHb_cyc2	82.4	94.9	92.5	86.7	0.97
ER, tHb, %tHb_cyc1	81.8	88.1	85.8	85.1	0.90
TN, tHb, %tHb_cyc1	82.0	88.1	85.9	85.1	0.90
tHb, %tHb_cyc1	83.8	88.1	86.1	86.5	0.95

*AUC* area under the curve, *ER* estrogen receptor, *HER2* human epidermal growth factor receptor 2, *NPV* negative predictive value, *PPV* positive predictive value, *tHb* total hemoglobin, *TN* triple negative

The %US ratio, the largest dimensions of post-treatment US measurements normalized to the pretreatment, of grade 1–3 and 4–5 tumors were calculated for 19 patients with well-defined US images. For grade 1–3 tumors (*n* = 9), %USs were 90.1 ± 9.8%, 84.9 ± 17.3%, and 77.0 ± 20% measured at end of cycles 1 to 3, respectively, whereas for grade 4–5 tumors (*n* = 11), %USs were 61.2 ± 18.0%, 52.3 ± 15.6%, and 42.8 ± 13.7%, respectively. Statistical significance was achieved at all cycles (*P* < 0.001, *P* < 0.001, and *P* = 0.001, respectively).

## Discussion

The clinical inclination to select patients for NAC who are more likely to be good responders accounts for the large number (11/22, 50%) of HER2^+^ tumors in this study, especially with the ability to utilize dual HER2 blockade in this setting. In the HER2^+^ group, there were Miller-Payne grade 4 and 5 responses in 91% despite the large number of cases that coexpressed ER. This apparent anomaly of HER2/ER coexpression is partially explained by the relatively low-level ER expression in two of the eight HER2^+^/ER^+^ cases. The remaining patients were either ER^+^/HER2^–^ or TN and had Miller-Payne 4 or 5 responses of 17% and 40%, respectively.

In our previous work, we dichotomized our comparison groups as pCR and near pCR (Miller-Payne grades 4–5) versus “other” (Miller-Payne grades 1–3). Our rationale for using these same comparison groups in this report is further supported as follows. In the original study by Ogston [36], the Miller-Payne 5 and 4 groups tended to track together with regard to 5-year disease-free survival after NAC and surgery (85% and 72%) versus 66%, 60%, and 55% for Miller-Payne 1–3, respectively. Later, Zhao et al. [42] while evaluating the Miller-Payne system using a different dataset found very similar 5-year distant disease-free survival and local recurrence-free survival rates for Miller-Payne 4–5 versus Miller-Payne 1–3. Finally, Symmans et al. [43] using the residual cancer burden (RCB) system and another separate dataset for evaluating tumor response after NAC also found that pCR and near-pCR had very similar survival curves after surgery (their categories RCB-0 and RCB-1 compared with RCB-2 (partial response) and RCB-3 (chemoresistant)).

In studies published to date, diffused optical tomography and diffused optical spectroscopy have demonstrated the potential for predicting breast cancer pathological response. Studies have shown that accurate predictions were made in the neoadjuvant setting by utilizing pretreatment hemoglobin levels or blood oxygen saturations (SO_2_) [20, 21, 23, 31], or by monitoring early changes of hemoglobin content and SO_2_ at 1 day or 1 week [19, 27], or after the first two cycles of NAC [20, 22, 23]. In this study, we have developed prediction models and have shown that the best pretreatment predictors are HER2 and tHb (AUC = 0.88). The pretreatment predictors based on ER and tHb, and TN and tHb predict response with moderate AUC accuracies of AUC 0.69 and 0.72, which are about the same as for the single predictor tHb (AUC = 0.75). For the HER2 subtype, the best window to accurately predict response is at the completion of the first two cycles of NAC. For ER or TN subtypes, the best window is at the completion of the first cycle of NAC and the best predictors are tHb and %tHb.

In our earlier study of 32 patients [21], the testing data obtained from cross-validations showed that the addition of the pretreatment tHb to pathological variables and biomarkers significantly improved the prediction (AUC 0.92 (95%CI 79.4–99.8)) compared with using these variables alone (AUC 0.84 (95% CI 57.2–99.0). The best pretreatment predictors of HER2, ER, and tHb reported in this study using the new cohort data as the testing set has achieved similar results of AUC 0.87 (95% CI 0.84–0.90).

NIR/US measurements obtained at the end of the first three treatment cycles were used for development and validation of the prediction models. Chemotherapy treatments are delivered generally in specific cycles. These schedules are based on maximal tumor cell kill and allowance of recovery of normal tissues (http://chemocare.com/chemotherapy/what-is-chemotherapy/cancer-cells-chemotherapy.aspx). Some cycles are given every 2 weeks and others every 3 weeks. Tumor responses by imaging studies occur after a specified number of treatment cycles, and not by specific times. Because treatments vary for HER2-positive disease compared with ER-positive or TN disease, the drugs utilized and schedules vary. The effects of the treatments are studied by equivalent treatment cycles. The goal was to give guideline-based treatments and to measure the maximal effect of each of these treatments before the next cycle is given. When combining all patients together, the measurements points were different in terms of weeks but not by cycles—they were consistent. The results show that pretreatment tHb and first cycle %tHb can achieve an accuracy of AUC 0.96 in the HER2 subtype, and AUCs of 0.91 in ER and 0.90 in TN subtypes, and AUC of 0.95 regardless of subtype. Thus, it is the ultimate effect of the drug on the tumor vascularity that is being assessed by near infrared functional parameters.

Baseline tHb measures tumor angiogenesis and correlates with tumor aggressiveness as evaluated by Spearman’s rhos with Nottingham score (rho = 0.355, *P* = 0.007) in the combined training and testing data of 54 patients. Aggressive tumors have high proliferative rates and respond quickly to chemotherapy, as shown by PET/CT which detects pretreatment and early changes in tumor metabolic activity after one or two cycles of NAC [44] and predicts pCR. As expected from log cell-kill kinetics of cytotoxic drugs, a given dose kills a constant proportion of a tumor cell population rather than a constant number of cells (https://bajan.files.wordpress.com/2010/09/principles-of-cancer-chemotherapy.pdf). Therefore, for chemosensitive tumors, there are more total cells killed in the first cycle of treatment and more tumor neovasculature damage that may cause a significant decrease in tumor hemoglobin measured by the NIR system. For HER2^+^ responders, the cycle 1 %tHb is lower (mean %tHb = 77%) and more predictive than that of ER^+^/HER2^–^ and TN responders (mean %tHb =84%) because trastuzumab has been demonstrated to have a strong antiangiogenic effect [45].

Our study has substantial implications for the combined use of tumor subtypes and NIR-measured tumor hemoglobin content in predicting pathological response even before therapy has begun. If a decision is made to initiate therapy, modification of the treatment can be implemented as soon as cycles 1 and 2 are completed, allowing for personalized treatment. This ability will be of even greater value as our armamentarium of interventions increases and responses can more effectively tailor the agents selected.

In this new cohort of patients, 86% of the tumors were visible on US compared with 65% in our earlier study [20]. This difference could be due to a greater representation of HER2-positive tumors in which US has been shown to be more accurate in measuring tumor size [46]. Pretreatment US does not predict response. However, %US of this cohort demonstrated statistical significance between responders and nonresponders at the early treatment cycles 1 to 3, while the earlier study group [20] showed no statistical significance at the end of cycles 1 and 2 (*P* = 0.437 and *P* = 0.172) between these two responder groups. Significance was achieved at the end of cycle 3. Additionally, earlier data showed no correlation between %US measured at the end of cycles 1 to 3 and Miller-Payne grade (rho = 0.27, *P* = 0.211; rho = 0.25, *P* = 0.257; and rho = 0.36, *P* = 0.106), respectively. With more patient data, the %US measure will be assessed on its role in predicting response.

There are some limitations to this study. First, all patients were referred for NAC after core biopsy, and hence the baseline NIR/US imaging was performed after the initial biopsy. Bruise or hematoma due to prior biopsy could have some effect on pretreatment NIR measurements. However, 21 patients had the pretreatment NIR measurements more than 10 days and one patient 7 days after biopsy. Based on the literature, the pretreatment NIR measurements 1 week after core biopsy were not affected by the biopsy [47]. However, another study followed a patient before and after biopsy and showed a 10% increase in diffuse optical spectroscopy (DOS)-measured deoxygenated hemoglobin after 9 days following biopsy [48]. Our study patient who imaged 7 days following biopsy had a pCR of Miller-Payne grade 5, and her cycle 1 %tHb was 58%, or a reduction of 42%. This level of %tHb change is too large to be counted as a biopsy effect. Secondly, this new pool of 22 patients had 50% HER2^+^ tumors and were treated with the dual HER2 blockade regimen which was not available in the training data obtained from 2008 to 2011. We have randomly selected data from 6 out of 11 patients from this study and added these 6 patients’ data to the training set to train the prediction models using all combinations. The testing data includes 73% from this study, with adequate samples of all subtypes. Thirdly, the training and testing datasets are still small and overfitting can occur when the training dataset is limited [21]. We have selected the minimal number of independent predictors for each prediction model, performed partial cross-validation, and used fairly high amounts (16/54, 30%) of the patient data for testing. The performances of the prediction models based on respective training and testing datasets are similar with no obvious pattern of higher AUC values for training data and much lower AUCs for testing data, which would be expected if there were problem of overfitting. With more patients recruited to the study, we will be able to establish a large database to validate prediction models with more input predictors.

The technical limitations of the US-guided NIR technique include the accuracy of the reconstructed optical absorption coefficients, the longitudinal repeatability of the measurements, and SO_2_ estimation. For a large high-contrast phantom of 3–5 cm in size, about 60–70% reconstruction accuracy in target absorption can be achieved [38]. Because the average pretreatment tumor sizes of the two responder groups were similar, any under-reconstruction should affect the light quantification of both groups similarly. Therefore, the comparison of pretreatment and early treatment hemoglobin levels between the two responder groups should be minimally affected. Additionally, because the same sized ROI obtained from the pretreatment US of each patient is used for reconstructions at all subsequent treatment cycles for the same patient, under-reconstruction should have a minimal effect on the %tHb, which is normalized to the pretreatment level. The longitudinal repeatability of the reconstructed phantom absorption coefficient is about 5–10%, which is obtained by repeatedly imaging solid absorbers embedded in the same concentration of Intralipid over a 1- to 2-year period. This level of change is much smaller than the changes seen in patients who responded to treatments. Finally, SO_2_ estimated from DOS has been reported as a good pretreatment predictor [31]. However, SO_2_ distribution = oxyHb distribution/tHb distribution is not as robust as tHb, oxyHb, and deoxyHb when the tHb values reconstructed from tomography are lower in some voxels, especially when the tumor is large and distribution is heterogeneous.

## Conclusions

In conclusion, our findings indicate that the breast tumor biomarkers (HER2, TN, and ER) combined with the pretreatment tumor total hemoglobin content are strong predictors of the response to NAC. The optimal treatment window to identify patients destined to have complete or near-complete responses is after the completion of the first two treatment cycles for HER2 tumors and the first treatment cycle for ER or TN tumors, when the assessment of total hemoglobin change is further predictive. This technology could be a valuable tool in personalizing treatments by response. These initial results remain to be validated with a larger trial of more patients.

## Abbreviations

AUC: Area under the ROC curve
CI: Confidence interval
CT: Computed tomography
deoxyHb: Deoxygenated hemoglobin
DOS: Diffuse optical spectroscopy
ER: Estrogen receptor
FISH: Fluorescence in situ hybridization
HER2: Human epidermal growth factor receptor 2
MRI: Magnetic resonance imaging
NAC: Neoadjuvant chemotherapy
NIR: Near infrared
NPV: Negative predictive value
NS: Nottingham histologic score
oxyHb: Oxygenated hemoglobin
pCR: Pathological complete response
PET: Positron emission tomography
PPV: Positive predictive value
PR: Progesterone receptor
RCB: Residual cancer burden
ROC: Receiver operating characteristic
ROI: Region of interest
SO_2_: Oxygen saturation
tHb: Total hemoglobin
TN: triple negative
US: Ultrasound
